# Prognostic Value of Blood Urea Nitrogen/Creatinine Ratio for Septic Shock: An Analysis of the MIMIC-III Clinical Database

**DOI:** 10.1155/2021/5595042

**Published:** 2021-05-22

**Authors:** Didi Han, Luming Zhang, Shuai Zheng, Fengshuo Xu, Chengzhuo Li, Rui Yang, Wen Ma, Haiyan Yin, Jun Lyu

**Affiliations:** ^1^Department of Clinical Research, The First Affiliated Hospital of Jinan University, Guangdong Province 510630, China; ^2^School of Public Health, Xi'an Jiaotong University Health Science Center, Shaanxi Province 710061, China; ^3^Intensive Care Unit, The First Affiliated Hospital of Jinan University, Guangzhou 510630, China; ^4^School of Public Health, Shaanxi University of Chinese Medicine, Xianyang, Shaanxi, China

## Abstract

**Background:**

Research has previously been done into the risk factors for mortality in septic shock patients. However, there has been no epidemiological study investigating the effect of the blood urea nitrogen/creatinine ratio (BCR) on the prognosis of critically ill septic shock patients. This study is aimed at determining the relationship between BCR and all-cause mortality in adult septic shock patients.

**Methods:**

Data were extracted from the MIMIC-III database. The clinical endpoints were 28-, 90-, and 365-day all-cause mortality rates in critically ill septic shock patients. Cox proportional hazards models and subgroup analyses were used to analyze the relationship between BCR quartiles and all-cause mortality in septic shock patients. Receiver operator characteristic (ROC) curves and areas under the ROC curves (AUCs) were calculated to evaluate how accurately BCR predicts the mortality of septic shock patients.

**Results:**

Among the 2484 septic shock patients extracted from the database, 619, 563, 677, and 625 fell into the first (<14.4 mg/dL), second (≥14.4 mg/dL and <20.0 mg/dL), third (≥20.0 mg/dL and <27.3 mg/dL), and fourth (≥27.3 mg/dL) quartiles of BCR, respectively. Male and white patients accounted for 53.8% (1336 patients) and 74.8% (1857 patients) of the population, respectively. The mean age of the population was 67.7 ± 15.8 years. An inverse M-shaped relationship between BCR and mortality in septic shock patients was identified, with a value of ≥27.3 mg/dL providing the highest risk (HR = 1.596, 95% CI: 1.396-1.824, *P* < 0.001). In the Cox regression model adjusted for different confounding variables, BCR values in the fourth quartiles were significantly associated with increased mortality, using the first quartiles as a reference. The areas under the ROC curves (AUCs) for BCR plus the Sequential Organ Failure Assessment (SOFA) score and BCR plus Acute Physiology Score III (APSIII) were 0.694 (95% CI: 0.673-0.716) and 0.724 (95% CI: 0.703-0.744), respectively.

**Conclusion:**

An inverse M-shaped curve was determined between BCR and the mortality of septic shock patients. BCR was identified as a readily available and independent prognostic biomarker for septic shock patients, and higher BCRs were associated with increased mortality in these patients.

## 1. Background

Sepsis is a syndrome of physiological, biochemical, and pathological abnormalities caused by infection [1]. Abnormal circulation and metabolism caused by septic shock can increase the mortality of intensive care unit (ICU) patients [2, 3]. The mortality rates of sepsis and septic shock have generally decreased, whereas the incidence rates have increased [4, 5]. Previous studies have indicated that severe sepsis and septic shock might cause 20–30% of all mortality and 30–50% of hospital mortality [6, 7].

Septic shock is currently defined as sepsis with hypotension, which requires vasopressor therapy to maintain a mean arterial pressure of 65 mmHg or higher. Despite adequate fluid resuscitation, serum lactic acid remains higher than 2 mmol/L [8]. The identification of risk factors that affect the prognosis of critically ill patients is helpful to guide medical workers and patients to take early intervention measures to reduce disease mortality [9]. Despite this, septic shock mortality remains high.

Blood urea nitrogen (BUN) and creatinine (Cr) can reflect the degree of damage to glomerular filtration function caused by external factors from the kidney. An increase in BUN often indicates the presence of a pathological condition, which is common in gastrointestinal bleeding. Intestinal bleeding results in more red blood cells being produced, and plasma proteins can be converted into a nitrogen source and absorbed into the blood. Clinically, Cr content is often used to detect changes in renal function, which helps to determine whether renal function is in a potential failure state or improved state. Many factors influence BUN and Cr levels [10, 11]. BUN is not a specific marker of renal insufficiency, and so predictions based on one of BUN or Cr alone may have limitations.

The BUN/Cr ratio (BCR) has recently been confirmed as a prognostic factor in patients with acute kidney injury, acute cerebral infarction, ischemic stroke, and acute decompensated heart failure [12–15]. However, no previous studies have determined the relationship between the BCR and the prognosis of septic shock patients. This retrospective cohort study is aimed at identifying the relationship between BCR and all-cause mortality in septic shock patients.

## 2. Materials and Methods

### 2.1. The MIMIC-III Database

This study included 2484 critically ill patients. The study data were extracted from the MIMIC-III database, which is a large, single-center, publicly available critical-care database [16]. It contains unconfirmed health-related data of more than 60,000 ICU patients between 2001 and 2012. The variables recorded in this database are demographics, vital signs, laboratory tests, medications, nursing progress records, and other related clinical variables. The MIMIC-III database was constructed by a collaborative research team at the Laboratory for Computational Physiology, Massachusetts Institute of Technology. To get access to the database, we complete the course “Protecting Human Research Participants” at the website of National Institutes of Health and obtained the certification (Record ID: 38292153) [17].

### 2.2. Study Population Selection and Data Extraction

Inclusion criteria included (1) patients over 18 years old, (2) patients diagnosed with septic shock according to the International Classification of Diseases 9 code (ICD-9) which is 785.52, and (3) patients who were hospitalized for the first time in the ICU for more than 2 days. Exclusion criteria included (1) BUN and Cr levels not measured during ICU hospitalization and (2) an individual data loss exceeding 5%. (More than 5% of the patient's clinical research information is missing.)

Demographics, vital signs, comorbidities, laboratory parameters, clinical severity scores, and other admission data were extracted. Comorbidities such as chronic lung, arrhythmia, coagulopathy, congestive heart failure (CHF), diabetes, electrolytes, hypertension, liver disease, and renal failure were also included in this study. Laboratory measurements were included for hemoglobin, hematocrit, platelets, prothrombin time (PT), partial thrombin time (PTT), red blood cell distribution width (RDW), lactate, international normal ratio (INR), anion gap, glucose, albumin, bicarbonate, bilirubin, sodium, potassium, calcium, BUN, Cr, white blood cell count (WBC), lymphocytes, and neutrophils. The Sequential Organ Failure Assessment (SOFA) score and Elixhauser score were extracted for each patient. Also extracted were age, sex, race, marital status, insurance status, admission type, mean blood pressure (MBP), temperature, heart rate, SpO_2_, respiratory rate, renal replacement therapy (RRT) use, mechanical ventilation use, hours of vasopressor use, and length of stay in the ICU. If the above laboratory parameters were tested multiple times within 24 hours, BCR is calculated according to the first test [18]. Patients were divided into four quartile groups based on the initial BCR value. The main endpoint was 28-day all-cause mortality, while the 90- and 365-day all-cause mortality rates were also study outcomes.

### 2.3. Statistical Analysis

The baseline characteristics of all patients were stratified according to BCR quartiles. Normally distributed continuous variables were reported as the means ± SD, whereas nonnormal variables were summarized as the median and interquartile range (IQR). Shapiro-Wilk tests were used to assess variable distributions. Categorical variables were expressed as frequencies and proportions and compared using chi-square tests and Fisher's exact test [19]. Log-rank testing was used to compare survival rates, and Kaplan-Meier curves were constructed [20]. Multivariate Cox proportional hazards regression was used to determine the relationship between BCR and 28-, 90-, and 365-day all-cause mortality rates. The results of these were expressed as hazard ratios (HRs) with 95% confidence intervals (CIs). Results were analyzed separately according to the cutoff value of BCRs obtained from a curve-fitting method [21].

We established two multivariate models to determine whether BCR is independently related to the outcome endpoint. In model I, covariates were only adjusted for age, sex, race, marital status, and insurance status [20]. In model II, we adjusted for age, sex, Elixhauser score, RRT use, mechanical ventilation use, chronic pulmonary, CHF, diabetes, fluid electrolyte, liver disease, renal failure, temperature, heart rate, hematocrit, platelets, glucose, SOFA score, MBP, SpO_2_, PT, lactate, albumin, bicarbonate, bilirubin, potassium, and calcium. We select these confounding factors based on the estimated impact of changes over 10%. Subgroup analysis of the associations between BCRs and 28-day all-cause mortality was performed using stratified linear regression models. Receiver operator characteristic (ROC) curve analysis is used to further evaluate the accuracy of BCRs [18].

All statistical analysis was performed using SPSS (version 21.0) and R software (version 3.6.1). Missing values were addressed using multiple imputation during Cox regression and model construction [22]. A two-tailed *P* value of <0.05 was considered statistically significant.

## 3. Results

### 3.1. Population and Baseline Characteristics

**Figure 1:**
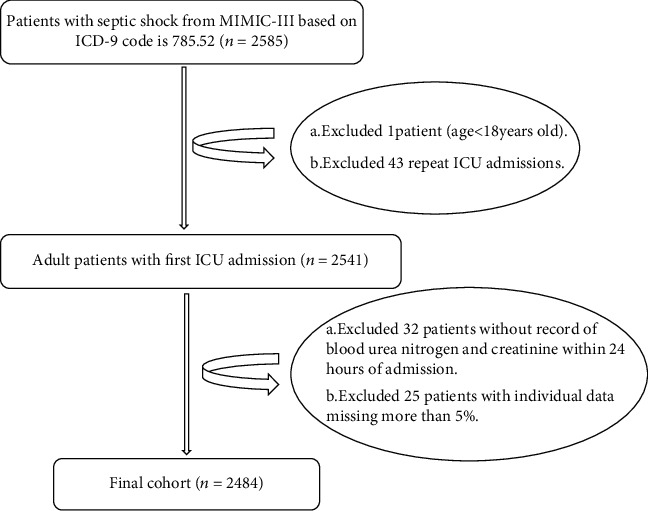
Flowchart of study patient selection.

Data included within 24 hours of admission from 2484 eligible septic shock patients were included in this study. The data selection procedure is displayed in Figure 1. Patient demographic characteristics stratified by BCR quartiles are listed in Table 1. The patients were aged 67.7 ± 15.8 years and comprised 1136 (53.8%) males and 1857 (74.8%) white patients. Emergency admission accounted for 2389 (96.2%) patients, 1617 (65.1%) had Medicare insurance, and 1774 (71.4%) were married. RRT was performed on 2253 (90.7%) patients, and 1419 (42.9%) were treated using mechanical ventilation.

According to BCR, 619, 563, 677, and 625 patients belonged to the first (<14.4 mg/dL), second (≥14.4 mg/dL and <20.0 mg/dL), third (≥20.0 mg/dL and <27.3 mg/dL), and fourth (≥27.3 mg/dL) quartiles, respectively. Patients with BCR ≥ 27.3 mg/dL were more likely to be female and elderly, were more likely to have comorbidities including CHF and fluid electrolyte imbalance, and had lower values of MBP, BMI, INR, heart rate, temperature, respiratory rate, anion gap, lactate, albumin, hematocrit, and hemoglobin and higher WBC, SOFA score, RDW, SpO_2_, sodium, potassium, calcium, bicarbonate, bilirubin, and mortality.

### 3.2. Association between BUN/Cr and Mortality

**Figure 2:**
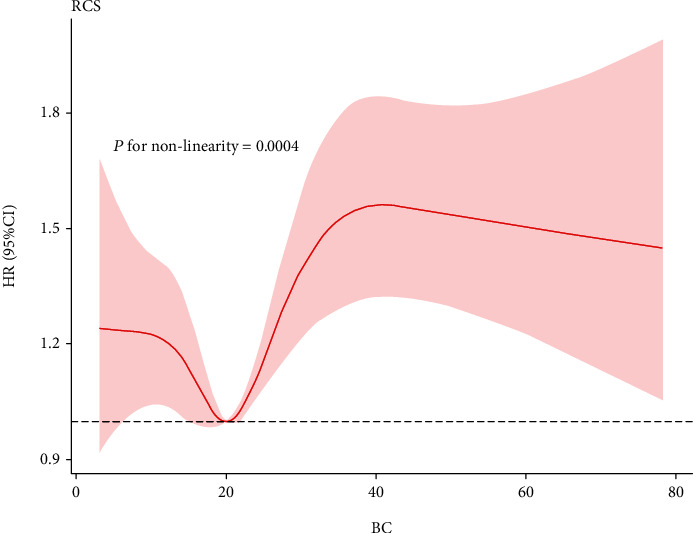
Nonparametric estimates of all-cause mortality on BCR among patients with septic shock.

**Figure 3:**
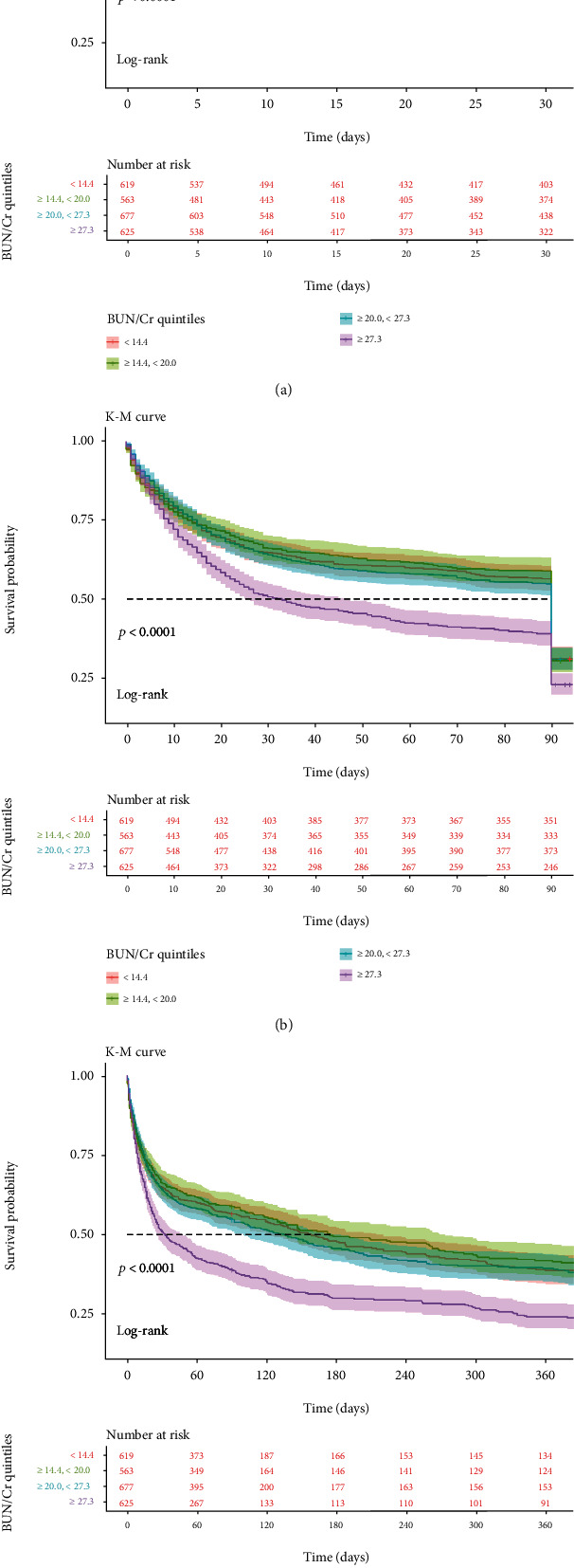
Kaplan-Meier curves showing the association between the BCR quartiles and all-cause mortality: (a) 28-day mortality; (b) 90-day mortality; (c) 365-day mortality.

The Cox proportional hazards regression model was used to determine the relationship between BCR and all-cause mortality in septic shock patients. We observed that BCR value and septic shock patient mortality had an M-shaped relationship (Figure 2). The Kaplan-Meier curve in Figure 3 displays the relationship between BCR quartiles and 28-, 90-, and 365-day mortality.

The first quartile BCR was used as a reference in model I and model II. In model I, higher BCR (fourth quartile vs. first quartile) was associated with increased risks of 28-, 90-, and 365-day all-cause mortality after adjusting for age, sex, race, marital status, and insurance status (HR = 1.281, 95%CI = 1.066–1.540, *P* = 0.008; HR = 1.385, 95%CI = 1.175–1.633, *P* < 0.001; and HR = 1.324, 95%CI = 1.139–1.540, *P* < 0.001, respectively). In model II, after adjustment for confounders including age, sex, Elixhauser score, RRT use, mechanical ventilation use, chronic pulmonary, CHF, diabetes, fluid electrolyte, liver disease, renal failure, temperature, heart rate, hematocrit, platelets, glucose, SOFA score, MBP, SpO_2_, PT, lactate, albumin, bicarbonate, bilirubin, potassium, and calcium, higher BCR was still significantly associated with 28-, 90-, and 365-day all-cause mortality rates (fourth quartile vs. first quartile: HR = 1.268, 95%CI = 1.037–1.551, *P* = 0.021; HR = 1.344, 95%CI = 1.124–1.606, *P* = 0.001; and HR = 1.309, 95%CI = 1.113–1.540, *P* = 0.001, respectively). For the purpose of sensitivity analysis, we also handled BCR as categorical variable (tertiles and quintiles) and found the same trend (*P* for trend: <0.0001). The results are listed in Table 2.

### 3.3. Subgroup Analysis

The association between different BCR levels and the 28-day all-cause mortality in septic shock patients was determined using a subgroup analysis. The significant interactions were PT (*P* = 0.007), MBP (*P* = 0.037), hours of vasopressor (*P* = 0.013), and platelets (*P* = 0.008). Patients who did not receive mechanical ventilation and had BCR > 27.3 had a higher risk of death at 28 days (HR: 3.467; 95% CI: 1.883–6.558; *P* < 0.001). Similarly, patients with temperature < 36.7°C, albumin < 2.7 g/dL, anion gap < 15, and RDW ≥ 15.4 showed an increased risk with a BCR ≥ 27.3 (HR, 95% CI, *P*: 1.612, 1.070–2.428; 1.538, 1.004–2.355; 1.700, 1.052–2.748; 1.678, 1.089–2.585, respectively). All results are presented in Table 3.

### 3.4. ROC Curve Analysis

The sensitivity and specificity of BCR and other variables (SOFA score, BCR plus SOFA score, APSIII, and BCR plus APSIII) were tested using ROC curves. Meanwhile, the area under the ROC curve (AUC) was calculated to evaluate the predictive performance of BCR for 28-, 90-, and 365-day all-cause mortality.

**Figure 4:**
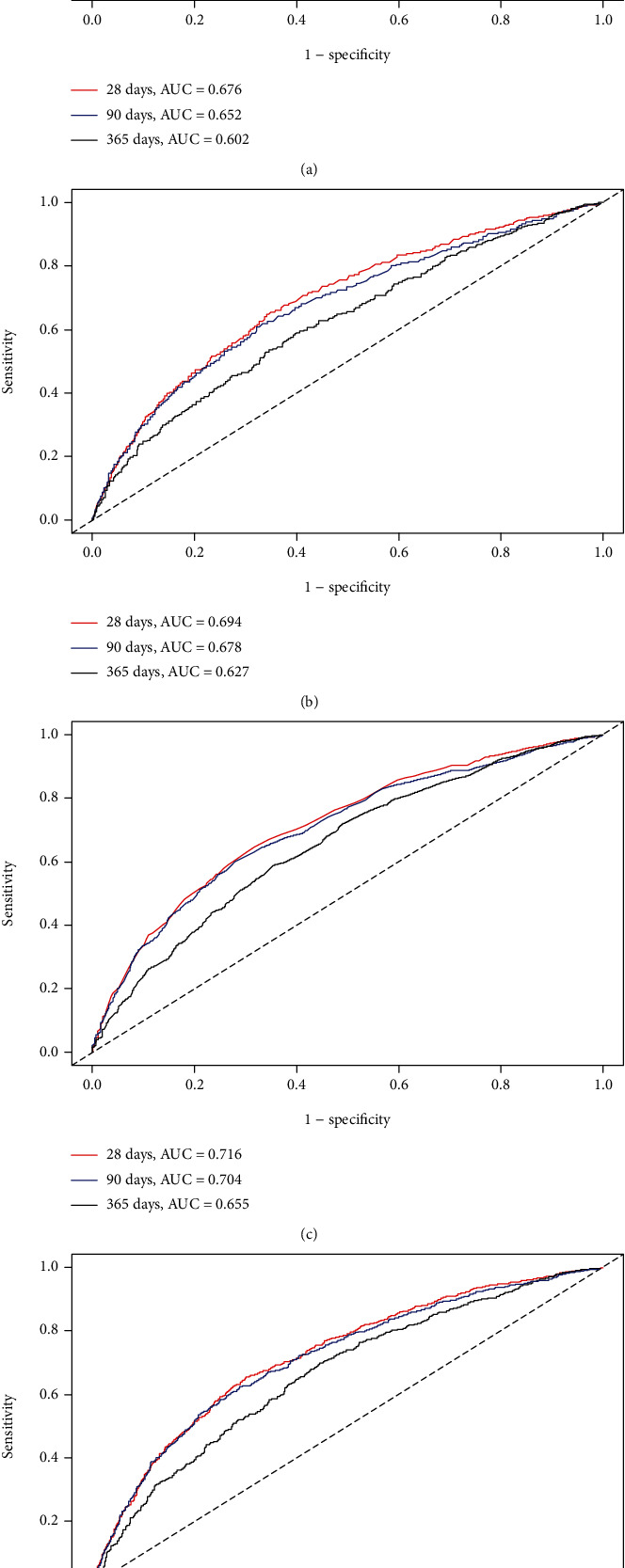
ROC curves for the prediction of mortality in critically ill patients with septic shock. (a, b) The ability of SOFA scores and BCR plus SOFA scores to predict 28-, 90-, and 365-day mortality. (c, d) The ability of APSIII scores and BCR plus APSIII scores to predict 28-, 90-, and 365-day mortality.

For the 28-day endpoint, the AUC was 0.676 (95% CI: 0.655-0.698) for the SOFA score, 0.694 (95% CI: 0.673-0.716) for BCR plus SOFA score, 0.716 (95% CI: 0.691-0.741) for APSIII, and 0.724 (95% CI: 0.703-0.744) for BCR plus APSIII. The generated ROC curves for 28-, 90-, and 365-day all-cause mortality are displayed in Figure 4.

## 4. Discussion

We studied 2484 septic shock patients to determine the relationships between BCR and 28-, 90-, and 365-day all-cause mortality rates. We found that compared with the preadmission first quartile (BCR < 14.4 mg/dL), high BCR (≥27.3 mg/dL) in septic shock patients within 24 hours of admission was significantly associated with all-cause mortality. After adjusting for age, sex, race, and other confounding factors, the study results remained reliable. Higher BCR was associated with an increase in mortality, suggesting that this ratio is a risk factor for the prognosis of septic shock patients. There was a nonlinear (M-shaped) relationship between BCR and mortality in critically ill septic shock patients [17]. To our knowledge, this is the first study of the relationship between BCR and all-cause mortality in septic shock patients.

Septic shock is a human body response to infection caused by abnormal circulation and cell metabolism [1]. The reported prevalence of sepsis is 12% of all ICU patients in the United States, and the hospital mortality rate for septic shock is approximately 40–60% [23]. Although the incidence of sepsis is decreasing every year, septic shock mortality rates remain very high [24].

BUN is known to be a risk factor for mortality in many cases, such as acute and chronic heart failure [25], coronary artery bypass grafts [26], acute pancreatitis [27], and bone marrow transplants [28]. BUN is also included in the general severity score of critically ill patients [29]. Changes in Cr concentration were directly related to glomerular filtration rate. When glomerular filtration function decreases, the concentration of serum Cr increases, indicating kidney damage, but this indicator has a low sensitivity, meaning that when it does rise, kidney function has been severely impaired [30]. Previous studies have found that BCR is a predictor of acute kidney injury and acute heart failure patient prognosis [31–34]. Takaya et al. [31–34] indicated that BCR ≥ 22 was associated with a poor survival prognosis in acute heart failure patients. Gastrointestinal bleeding can increase catabolism or increase urea absorption in the intestines, leading to increased BUN. This suggests that the higher BCR means a more severe condition.

When septic shock occurs, blood is affected by many inflammatory factors, which can lead to acute kidney injury and even acute renal failure. BUN and Cr levels are commonly used clinical indicators of renal function. Brisco et al. [35] also found that BCR and increased mortality are related, but there have been few studies showing that BCR is related to the prognosis of septic shock patients.

We found that a higher BCR was a risk factor for mortality in septic shock patients. The first quartile was the reference value, and the second and third quartiles had relatively low HR values and hence were protective factors. Regression adjusted for covariates found that the relationship between BCR and prognosis was M-shaped. And we did find that patients included in the group of the highest BCR (≥27.3 mg/dL) have significantly higher 28-day mortality compared to those of the other three groups. This may be because BCRs in the second and third quartiles are within the normal range, the patient's condition is normal, and the risk of death is low, whereas BCRs that are either too low or too high will increase the risk of death and lead to a poor prognosis. In general, there are two clinical reasons for BCR increasing. One is that only the increase of BUN will cause an increase in BCR when Cr is normal. This can occur during dehydration, blood loss, gastrointestinal bleeding [36], and eating a large amount of high-protein foods [10]. The other is prerenal oliguria, which occurs via a decrease in renal blood circulation, which leads to increases in Cr and BUN and a decrease in urine output. If BCR decreases, renal oliguria or postrenal oliguria might be present. Recent studies have indicated BCR ≥ 15 mg/dL as a precursor to dehydration, which may be related to complications from acute cerebral infarction and early deterioration of neurological function [37, 38]. However, the relationship between this indicator and septic shock has not yet been studied. It is worth noting that our results show a nonlinear relationship between BCR and the prognosis of patients with septic shock.

According to our results, higher BCR worsens the prognosis of septic shock patients, which is consistent with previous studies [35]. Referred to the first quartile, we found that the HR value of the fourth quantiles was greater than 1, suggesting that it was a risk factor and it was statistically significant. Clinicians should therefore note changes in renal function and BCR in septic shock patients in order to improve their prognosis.

The strengths of this study include it being the first time that the relationship between BCR and all-cause mortality has been investigated in septic shock patients based on a large and diverse population from a public database (the MIMIC-III database), which increases the significance of our research results. In addition, after adjusting for several confounding factors, multiple Cox regression analyses were performed and the relationship between BCR and all-cause mortality was still observed, indicating the good stability of our results. Since BCR is the basic index of clinical blood routine, the parameters are simple to collect, and our research results can be used to support other death indexes and improve prognosis prediction accuracy for septic shock patients [35].

This study also had some limitations. Firstly, this study used data from a publicly accessible single-center database, which questions of the generalizability of our conclusions, as well as confounding variables caused by missing data. However, this database has been utilized by many researchers globally, with the published articles improving the data quality, potentially improving the generalization of our findings. Secondly, BUN and Cr were measured only when the patient entered the ICU, without laboratory follow-up data. The measurement data may be classified incorrectly, which may affect the summary results. Thirdly, excluding patients who have not measured BUN and Cr values may cause sample selection bias. Finally, the database is relatively old, with patient information available between 2001 and 2012. Some information on septic shock diseases has changed recently, which may also affect the generalizability of our research results.

## 5. Conclusion

Our research indicates that there is a nonlinear (M-shaped) relationship between BCR and all-cause mortality. Higher quartile BCR values will increase the 28-, 90-, and 365-day all-cause mortality rates of septic shock patients and can better guide the risk stratification of critically ill patients in clinical practice. The present findings need to be validated through further large-scale prospective studies and longer follow-ups.

## Figures and Tables

**Table 1:** Characteristics of the study patients according to BCR.

Variables		BCR				*P*
		<14.4	≥14.4, <20.0	≥20.0, <27.3	≥27.3	
Total	*N* = 2484	*N* = 619	*N* = 563	*N* = 677	*N* = 625	
Gender, *n* (%)						0.016
Male	1336 (53.8)	364 (58.8)	302 (53.6)	358 (52.9)	312 (49.9)	
Female	1148 (46.2)	255 (41.2)	261 (46.4)	319 (47.1)	313 (50.1)	
Ethnicity, *n* (%)						
White	1857 (74.8)	388 (62.7)	445 (79.0)	525 (77.5)	499 (79.8)	<0.001
Black	262 (10.5)	130 (21.0)	39 (6.93)	50 (7.39)	43 (6.88)	
Other	365 (14.7)	101 (16.3)	79 (14.0)	102 (15.1)	83 (13.3)	
Insurance, *n* (%)						
Government	47 (1.9)	21 (3.39)	11 (1.95)	8 (1.18)	7 (1.12)	
Medicaid	192 (7.7)	68 (11.0)	35 (6.22)	43 (6.35)	46 (7.36)	
Medicare	1617 (65.1)	343 (55.4)	350 (62.2)	474 (70.0)	450 (72.0)	<0.001
Private	618 (24.9)	184 (29.7)	165 (29.3)	148 (21.9)	121 (19.4)	
Self-pay	10 (0.4)	3 (0.48)	2 (0.36)	4 (0.59)	1 (0.16)	
Marital status, *n* (%)						
Married	1774 (71.4)	400 (64.6)	405 (71.9)	515 (76.1)	454 (72.6)	<0.001
Unmarried	690 (27.8)	216 (34.9)	151 (26.8)	154 (22.7)	169 (27.0)	
Other	20 (0.80)	3 (0.48)	7 (1.24)	8 (1.18)	2 (0.32)	
RRT, *n* (%)						<0.001
Yes	2253 (90.7)	456 (73.7)	527 (93.6)	663 (97.9)	607 (97.1)	
No	231 (9.3)	163 (26.3)	36 (6.39)	14 (2.07)	18 (2.88)	
Mech. vent, *n* (%)						0.108
Yes	1065 (42.9)	283 (45.7)	241 (42.8)	297 (43.9)	244 (39.0)	
No	1419 (57.1)	336 (54.3)	322 (57.2)	380 (56.1)	381 (61.0)	
Chronic pulmonary, *n* (%)						0.799
Yes	1889 (76.0)	472 (76.3)	434 (77.1)	506 (74.7)	477 (76.3)	
No	595 (24.0)	147 (23.7)	129 (22.9)	171 (25.3)	148 (23.7)	
Cardiac arrhythmias, *n* (%)						0.961
Yes	1418 (58.1)	355 (57.4)	323 (57.4)	389 (57.5)	351 (56.2)	
No	1066 (42.9)	264 (42.6)	240 (42.6)	288 (42.5)	274 (43.8)	
Coagulopathy, *n* (%)						0.924
Yes	1710 (69.8)	425 (68.7)	394 (70.0)	462 (68.2)	429 (68.6)	
No	774 (31.2)	194 (31.3)	169 (30.0)	215 (31.8)	196 (31.4)	
CHF, *n* (%)						0.731
Yes	1533 (61.7)	373 (60.3)	344 (61.1)	421 (62.2)	395 (63.2)	
No	951 (38.3)	246 (39.7)	219 (38.9)	256 (37.8)	230 (36.8)	
Diabetes, *n* (%)						0.174
Yes	1883 (75.8)	474 (76.6)	440 (78.2)	514 (75.9)	455 (72.8)	
No	601 (24.2)	145 (23.4)	123 (21.8)	163 (24.1)	170 (27.2)	
Fluid electrolyte, *n* (%)						0.498
Yes	1034 (41.6)	258 (41.7)	243 (43.2)	266 (39.3)	267 (42.7)	
No	1450 (58.4)	361 (58.3)	320 (56.8)	411 (60.7)	358 (57.3)	
Hypertension, *n* (%)						0.526
Yes	1171 (47.1)	294 (47.5)	279 (49.6)	314 (46.4)	284 (45.4)	
No	1313 (52.9)	325 (52.5)	284 (50.4)	363 (53.6)	341 (54.6)	
Liver disease, *n* (%)						0.628
Yes	1942 (79.2)	477 (77.1)	446 (79.2)	537 (79.3)	482 (77.1)	
No	542 (21.8)	142 (22.9)	117 (20.8)	140 (20.7)	143 (22.9)	
Renal failure, *n* (%)						0.562
Yes	1868 (75.2)	465 (75.1)	416 (73.9)	522 (77.1)	465 (74.4)	
No	616 (24.8)	154 (24.9)	147 (26.1)	155 (22.9)	160 (25.6)	
Age (year)	67.7 (15.8)	60.7 (15.7)	68.2 (15.1)	70.5 (15.2)	71.0 (14.9)	<0.001
PT (second)	18.7 (12.1)	19.6 (13.2)	18.6 (10.6)	18.3 (12.2)	18.5 (12.3)	0.184
PTT (second)	38.0 (21.2)	40.0 (22.6)	37.9 (20.6)	37.2 (21.0)	36.9 (20.4)	0.039
INR	1.88 (2.12)	2.05 (3.14)	1.84 (1.39)	1.81 (1.76)	1.82 (1.73)	0.123
RDW	16.0 (2.43)	16.1 (2.45)	15.6 (2.24)	15.8 (2.37)	16.6 (2.55)	<0.001
SpO_2_ (%)	96.4 (4.18)	96.1 (4.94)	96.3 (3.97)	96.5 (3.48)	96.6 (4.24)	0.253
Temperature (°C)	36.8 (0.83)	36.9 (0.85)	36.8 (0.86)	36.8 (0.80)	36.7 (0.80)	<0.001
BMI (kg/m^2^)	28.5 (8.21)	29.2 (8.34)	29.5 (8.45)	28.2 (8.16)	27.3 (7.75)	<0.001
WBC (10^9/^L)	14.0 (10.3)	13.7 (9.35)	13.6 (9.85)	14.2 (11.6)	14.3 (9.95)	0.611
SOFA	7.82 (3.92)	8.52 (3.98)	7.77 (3.94)	7.61 (3.97)	7.42 (3.70)	<0.001
Glucose (mg/dL)	144.5 (52.9)	141 (51.6)	145 (57.4)	147 (54.5)	145 (47.8)	0.251
Heart rate (beats/minute)	92.5 (17.9)	93.5 (18.2)	92.3 (18.1)	92.5 (17.4)	91.6 (17.9)	0.315
MBP (mmHg)	70.98 (9.18)	71.7 (10.0)	70.9 (8.54)	71.0 (8.74)	70.3 (9.26)	0.077
Respiratory rate (beats/minute)	21.4 (4.72)	21.3 (4.83)	21.5 (4.35)	21.7 (4.85)	21.3 (4.78)	0.292
Albumin (g/dL)	2.82 (0.68)	2.90 (0.70)	2.85 (0.66)	2.85 (0.66)	2.69 (0.67)	<0.001
Bilirubin (mg/dL)	1.98 (4.12)	2.02 (3.81)	1.73 (3.46)	1.82 (3.96)	2.34 (5.02)	0.048
Bicarbonate (mg/dL)	22.3 (5.69)	22.1 (5.61)	21.4 (5.25)	21.9 (5.39)	23.7 (6.22)	<0.001
Calcium (mmol/L)	8.10 (1.09)	8.03 (1.16)	8.03 (1.05)	8.17 (1.06)	8.18 (1.10)	0.010
Potassium (mmol/L)	4.41 (1.00)	4.31 (1.06)	4.37 (0.94)	4.43 (0.96)	4.52 (1.02)	0.002
Sodium (mmol/L)	137.57 (6.89)	137 (5.50)	137 (6.14)	137 (6.75)	139 (8.61)	<0.001
Hematocrit (%)	33.75 (6.45)	34.3 (6.39)	34.3 (6.26)	33.7 (6.37)	32.7 (6.65)	<0.001
Hemoglobin (g/dL)	11.11 (2.18)	11.2 (2.23)	11.3 (2.11)	11.2 (2.14)	10.7 (2.22)	<0.001
Platelet (10^9/^L)	239 (153)	223 (138)	246 (146)	245 (162)	243 (161)	0.022
Lactate (mmol/L)	3.12 (2.38)	3.48 (2.97)	3.29 (2.43)	3.07 (2.14)	2.69 (1.79)	<0.001
Anion gap (mmol/L)	17.32 (5.08)	18.4 (5.49)	17.6 (5.09)	17.3 (4.81)	16.0 (4.62)	<0.001
Lymphocytes (%)	10.6 (11.9)	10.5 (10.6)	11.0 (12.3)	10.7 (12.9)	10.3 (11.7)	0.780
Neutrophils (%)	76.9 (18.2)	76.7 (17.3)	76.0 (18.0)	76.9 (19.1)	77.7 (18.2)	0.456
Hours of vasopressor	67.5 (96.2)	72.1 (103)	65.3 (100)	62.5 (90.4)	70.5 (91.4)	0.250
ICU LOS (day)	7.76 (9.71)	7.61 (9.44)	7.91 (10.14)	7.20 (8.49)	8.40 (10.72)	0.154
Hospital LOS (day)	7.75 (9.70)	7.58 (9.44)	7.90 (10.14)	7.18 (8.49)	8.39 (10.73)	0.148
28-day mortality, *n* (%)	936 (37.7)	215 (34.7)	183 (32.5)	237 (35.0)	301 (48.2)	<0.001
90-day mortality, *n* (%)	1757 (70.7)	424 (68.5)	389 (69.1)	465 (68.7)	479 (76.6)	0.003
365-day mortality, *n* (%)	1983 (79.8)	485 (78.4)	440 (78.2)	524 (77.4)	534 (85.4)	0.001

BCR: blood urea nitrogen/creatinine ratio; RRT: renal replacement therapy; CHF: congestive heart failure; PT: prothrombin time; PTT: partial thrombin time; RDW: red blood cell distribution width; INR: international normal ratio; BMI: body mass index; WBC: white blood cell count; SOFA: Sequential Organ Failure Assessment score; MBP: mean blood pressure; ICU: intensive care unit; LOS: length of stay.

**Table 2:** HRs (95% CIs) for all-cause mortality across groups of BCR.

Variables	Nonadjusted			Model I			Model II		
	HR	95% CI	*P* value	HR	95% CI	*P* value	HR	95% CI	*P* value
28-day all-cause mortality									
BCR	1.011	1.006-1.016	<0.001	1.009	1.004-1.014	<0.001	1.007	1.002-1.013	0.013
Tertiles									
<16.3	Reference			Reference			Reference		
≥16.3, <24.3	1.032	0.875-1.218	0.708	0.911	0.768-1.081	0.286	0.990	0.827-1.186	0.913
≥24.3	1.390	1.190-1.624	<0.001	1.220	1.037-1.436	0.016	1.186	0.993-1.416	0.060
*P* for trend	<0.0001			<0.0001			<0.0001		
Quartiles									
<14.4	Reference			Reference			Reference		
≥14.4, <20.0	0.937	0.769-1.141	0.516	0.842	0.688-1.031	0.096	0.969	0.785-1.195	0.766
≥20.0, <27.3	0.996	0.828-1.198	0.969	0.862	0.710-1.042	0.124	0.942	0.767-1.157	0.571
≥27.3	1.485	1.246-1.769	<0.001	1.281	1.066-1.540	0.008	1.268	1.037-1.551	0.021
*P* for trend	<0.0001			<0.0001			<0.0001		
Quintiles									
<13.3	Reference			Reference			Reference		
≥13.3, <17.7	0.810	0.652-1.004	0.055	0.744	0.597-0.928	0.009	0.903	0.718-1.136	0.385
≥17.7, <22.5	0.911	0.738-1.124	0.385	0.775	0.623-0.963	0.022	0.996	0.791-1.253	0.970
≥22.5, <30.0	1.036	0.846-1.270	0.731	0.879	0.711-1.087	0.233	0.921	0.733-1.156	0.477
≥30.0	1.403	1.158-1.699	<0.001	1.203	0.984-1.471	0.071	1.247	1.002-1.552	0.047
*P* for trend	<0.0001			<0.0001			<0.0001		
90-day all-cause mortality									
BCR	1.013	1.009-1.017	<0.001	1.012	1.008-1.016	<0.001	1.009	1.005-1.014	<0.001
Tertiles									
<16.3	Reference			Reference			Reference		
≥16.3, <24.3	1.026	0.886-1.189	0.729	0.913	0.784-1.063	0.241	0.981	0.836-1.151	0.812
≥24.3	1.452	1.265-1.666	<0.001	1.285	1.113-1.484	<0.001	1.240	1.060-1.450	0.007
*P* for trend	<0.0001			<0.0001			<0.0001		
Quartiles									
<14.4	Reference			Reference			Reference		
≥14.4, <20.0	0.939	0.787-1.119	0.481	0.858	0.716-1.028	0.097	0.969	0.804-1.167	0.738
≥20.0, <27.3	1.036	0.880-1.221	0.670	0.904	0.763-1.073	0.249	0.984	0.821-1.179	0.856
≥27.3	1.575	1.347-1.841	<0.001	1.385	1.175-1.633	<0.001	1.344	1.124-1.606	0.001
*P* for trend	<0.0001			<0.0001			<0.0001		
Quintiles									
<13.3	Reference			Reference			Reference		
≥13.3, <17.7	0.853	0.705-1.033	0.104	0.797	0.655-0.969	0.023	0.942	0.770-1.153	0.564
≥17.7, <22.5	0.906	0.750-1.095	0.308	0.784	0.644-0.954	0.015	0.977	0.796-1.200	0.824
≥22.5, <30.0	1.073	0.894-1.288	0.447	0.921	0.761-1.115	0.400	0.967	0.789-1.184	0.742
≥30.0	1.552	1.309-1.841	<0.001	1.358	1.135-1.626	<0.001	1.377	1.134-1.671	0.001
*P* for trend	<0.0001			<0.0001			<0.0001		
365-day all-cause mortality									
BCR	1.012	1.009-1.016	<0.001	1.010	1.007-1.014	<0.001	1.008	1.004-1.013	<0.001
Tertiles									
<16.3	Reference			Reference			Reference		
≥16.3, <24.3	1.002	0.894-1.168	0.749	0.899	0.782-1.032	0.131	0.956	0.827-1.105	0.541
≥24.3	1.415	1.247-1.606	<0.001	1.243	1.089-1.148	<0.001	1.212	1.051-1.397	0.008
*P* for trend	<0.0001			<0.0001			<0.0001		
Quartiles									
<14.4	Reference			Reference			Reference		
≥14.4, <20.0	0.938	0.800-1.100	0.432	0.848	0.721-1.000	0.049	0.957	0.810-1.133	0.616
≥20.0, <27.3	1.011	0.872-1.174	0.881	0.874	0.748-1.021	0.089	0.945	0.802-1.112	0.494
≥27.3	1.526	1.323-1.761	<0.001	1.324	1.139-1.540	<0.001	1.309	1.113-1.540	0.001
*P* for trend	<0.0001			<0.0001			<0.0001		
Quintiles									
<13.3	Reference			Reference			Reference		
≥13.3, <17.7	0.865	0.728-1.029	0.102	0.795	0.666-0.950	0.012	0.928	0.773-1.114	0.422
≥17.7, <22.5	0.934	0.786-1.106	0.423	0.797	0.667-0.952	0.013	0.975	0.810-1.172	0.784
≥22.5, <30.0	1.029	0.870-1.217	0.737	0.869	0.728-1.036	0.118	0.905	0.752-1.089	0.291
≥30.0	1.535	1.312-1.796	<0.001	1.326	1.124-1.564	<0.001	1.364	1.143-1.628	<0.001
*P* for trend	<0.0001			<0.0001			<0.0001		

HR: hazard ratio; CI: confidence interval. Models were derived from Cox proportional hazards regression models. Nonadjusted model adjusted for none. Adjust I model adjusted for age, sex, race, marital status, and insurance status. Adjust II model adjusted for age, sex, Elixhauser score, RRT use, mechanical ventilation use, chronic pulmonary, CHF, diabetes, fluid electrolyte, liver disease, renal failure, temperature, heart rate, hematocrit, platelets, glucose, SOFA score, MBP, SpO_2_, PT, lactate, albumin, bicarbonate, bilirubin, potassium, and calcium.

**Table 3:** Subgroup analysis of the associations between the BCR and 28-day all-cause mortality.

Characteristics	No. of patients	BCR				*P* for interaction
		<14.4	≥14.4, <20.0	≥20.0, <27.3	≥27.3	
		HR (95% CI)	HR (95% CI)	HR (95% CI)	HR (95% CI)	
Age (year)						0.104
<69	1228	1.0 (ref)	0.824 (0.590-1.152)	0.930 (0.652-1.326)	1.165 (0.709-1.914)	
≥69	1256	1.0 (ref)	1.130 (0.828-1.544)	0.900 (0.656-1.236)	1.275 (0.858-1.896)	
Gender						0.867
Male	1336	1.0 (ref)	0.831 (0.616-1.121)	0.853 (0.630-1.156)	0.936 (0.611-1.433)	
Female	1148	1.0 (ref)	1.033 (0.733-1.454)	0.849 (0.591-1.220)	1.206 (0.770-1.889)	
Ethnicity						0.666
White	1857	1.0 (ref)	0.974 (0.753-1.261)	0.886 (0.679-1.155)	0.886 (0.679-1.155)	
Black	262	1.0 (ref)	0.843 (0.341-2.041)	0.482 (0.191-1.214)	0.846 (0.236-3.037)	
Other	365	1.0 (ref)	0.695 (0.381-1.266)	0.945 (0.498-1.791)	0.631 (0.249-1.600)	
Marital status						0.980
Married	1774	1.0 (ref)	0.907 (0.700-1.175)	0.846 (0.629-1.102)	1.144 (0.810-1.616)	
Unmarried	690	1.0 (ref)	0.907 (0.566-1.454)	0.866 (0.527-1.423)	1.160 (0.594-2.264)	
Other	20	1.0 (ref)	—	—	—	
RRT						0.065
Yes	231	1.0 (ref)	0.829 (0.398-1.726)	0.585 (0.130-2.263)	0.714 (0.147-3.465)	
No	2253	1.0 (ref)	1.050 (0.822-1.341)	0.982 (0.769-1.255)	1.255 (0.913-1.724)	
Mech. vent						0.484
Yes	1419	1.0 (ref)	0.884 (0.684-1.143)	0.812 (0.620-1.064)	0.812 (0.620-1.064)	
No	1065	1.0 (ref)	1.817 (1.111-2.973)	1.732 (1.054-2.847)	3.467 (1.833-6.558)	
SOFA						0.955
<5	746	1.0 (ref)	1.053 (0.578-1.919)	0.910 (0.505-1.640)	1.240 (0.599-2.564)	
≥5	1738	1.0 (ref)	0.946 (0.743-1.204)	0.881 (0.685-1.134)	1.155 (0.818-1.632)	
Elixhauser score						0.450
<13	1154	1.0 (ref)	0.665 (0.458-0.964)	0.623 (0.422-0.921)	0.916 (0.537-1.561)	
≥13	1330	1.0 (ref)	1.150 (0.871-1.518)	1.066 (0.800-1.420)	1.406 (0.963-2.051)	
PT (second)						0.007
<14.9	1213	1.0 (ref)	0.909 (0.604-1.366)	0.943 (0.637-1.397)	1.619 (0.995-2.635)	
≥14.9	1271	1.0 (ref)	0.992 (0.759-1.298)	0.826 (0.642-1.157)	0.964 (0.637-1.460)	
PTT (second)						0.535
<32	1240	1.0 (ref)	1.073 (0.742-1.550)	0.965 (0.669-1.391)	1.374 (0.866-2.178)	
≥32	1244	1.0 (ref)	0.883 (0.665-1.173)	0.813 (0.600-1.101)	1.006 (0.662-1.528)	
SpO_2_ (%)						0.528
<97	1212	1.0 (ref)	0.894 (0.658-1.214)	0.742 (0.532-1.034)	1.231 (0.788-1.924)	
≥97	1272	1.0 (ref)	0.976 (0.702-1.357)	0.977 (0.704-1.355)	1.043 (0.680-1.600)	
Temperature (°C)						0.118
<36.7	1189	1.0 (ref)	1.441 (1.057-1.964)	1.087 (0.791-1.495)	1.612 (1.070-2.428)	
≥36.7	1295	1.0 (ref)	0.657 (0.474-0.910)	0.764 (0.546-1.069)	0.879 (0.552-1.402)	
Heart rate (beats/minute)						0.291
<91.4	1242	1.0 (ref)	1.008 (0.723-1.407)	0.777 (0.547-1.104)	1.335 (0.849-2.101)	
≥91.4	1242	1.0 (ref)	0.904 (0.671-1.220)	0.978 (0.722-1.324)	0.985 (0.651-1.489)	
MBP (mmHg)						0.037
<70.1	1234	1.0 (ref)	1.125 (0.837-1.512)	0.854 (0.622-1.172)	1.422 (0.932-2.171)	
≥70.1	1250	1.0 (ref)	0.816 (0.579-1.151)	0.958 (0.684-1.341)	0.928 (0.587-1.466)	
Respiratory rate (beats/minute)						0.428
<21.4	1336	1.0 (ref)	0.916 (0.667-1.257)	0.832 (0.598-1.159)	1.482 (0.966-2.274)	
≥21.4	1148	1.0 (ref)	0.989 (0.722-1.355)	0.825 (0.595-1.145)	0.891 (0.562-1.411)	
Glucose (mg/dL)						0.624
133.9	1242	1.0 (ref)	0.971 (0.710-1.327)	1.078 (0.781-1.488)	1.497 (0.982-2.281)	
≥133.9	1242	1.0 (ref)	0.867 (0.626-1.199)	0.686 (0.492-0.957)	0.808 (0.514-1.268)	
Albumin (g/dL)						0.451
<2.7	1194	1.0 (ref)	1.171 (0.860-1.595)	1.083 (0.781-1.500)	1.538 (1.004-2.355)	
≥2.7	1290	1.0 (ref)	0.826 (0.594-1.148)	0.751 (0.542-1.041)	1.008 (0.659-1.541)	
Bilirubin (mg/dL)						0.555
<0.7	1229	1.0 (ref)	0.965 (0.676-1.378)	0.866 (0.601-1.246)	1.173 (0.734-1.874)	
≥0.7	1255	1.0 (ref)	0.935 (0.702-1.245)	0.848 (0.625-1.151)	1.031 (0.679-1.564)	
Bicarbonate (mg/dL)						0.312
<22	1072	1.0 (ref)	0.965 (0.699-1.332)	1.012 (0.712-1.438)	1.032 (0.615-1.731)	
≥22	1412	1.0 (ref)	0.937 (0.612-1.288)	0.811 (0.593-1.109)	1.256 (0.856-1.841)	
Calcium (mmol/L)						0.981
<8.1	1172	1.0 (ref)	0.971 (0.700-1.346)	0.891 (0.618-1.285)	1.197 (0.717-1.997)	
≥8.1	1312	1.0 (ref)	0.899 (0.663-1.217)	0.826 (0.611-1.117)	1.116 (0.764-1.630)	
Potassium (mmol/L)						0.231
<4.2	1107	1.0 (ref)	0.860 (0.602-1.228)	0.888 (0.614-1.285)	1.280 (0.759-2.160)	
≥4.2	1377	1.0 (ref)	1.053 (0.789-1.406)	0.855 (0.635-1.151)	1.097 (0.753-1.600)	
Sodium (mmol/L)						0.098
<13.8	1222	1.0 (ref)	0.877 (0.641-1.200)	0.765 (0.555-1.055)	1.013 (0.652-1.573)	
≥13.8	1262	1.0 (ref)	1.016 (0.737-1.402)	0.950 (0.681-1.325)	1.306 (0.848-2.011)	
Hematocrit (%)						0.329
<33.3	1234	1.0 (ref)	0.935 (0.679-1.285)	0.984 (0.712-1.360)	1.337 (0.884-2.023)	
≥33.3	1250	1.0 (ref)	0.933 (0.681-1.277)	0.769 (0.550-1.074)	0.928 (0.592-1.455)	
Anion gap						0.268
<15	1001	1.0 (ref)	0.935 (0.649-1.349)	1.234 (0.856-1.777)	1.700 (1.052-2.748)	
≥15	1483	1.0 (ref)	0.981 (0.734-1.311)	0.797 (0.588-1.080)	1.023 (0.690-1.515)	
Lymphocytes (%)						0.712
<7.1	1242	1.0 (ref)	0.837 (0.607-1.154)	0.881 (0.635-1.222)	1.095 (0.709-1.693)	
≥7.1	1242	1.0 (ref)	1.000 (0.730-1.370)	0.855 (0.613-1.193)	1.171 (0.770-1.781)	
Neutrophils (%)						0.308
<81.9	1219	1.0 (ref)	0.957 (0.684-1.340)	0.902 (0.641-1.270)	1.357 (0.874-2.107)	
≥81.9	1265	1.0 (ref)	0.939 (0.697-1.265)	0.914 (0.666-1.254)	1.113 (0.719-1.723)	
Hours of vasopressor						0.013
<34	1223	1.0 (ref)	0.989 (0.693-1.413)	0.739 (0.506-1.081)	1.268 (0.760-2.117)	
≥34	1261	1.0 (ref)	1.057 (0.872-1.554)	1.164 (0.872-1.554)	1.306 (0.894-1.910)	
Platelet (10^9^/L)						0.008
<214	1241	1.0 (ref)	0.782 (0.567-1.079)	0.824 (0.595-1.142)	1.312 (0.853-2.017)	
≥214	1243	1.0 (ref)	1.062 (0.774-1.457)	0.888 (0.641-1.233)	0.962 (0.617-1.501)	
Hemoglobin (g/dL)						0.537
<10.8	1204	1.0 (ref)	0.665 (0.462-0.958)	0.638 (0.437-0.931)	0.931 (0.555-1.560)	
≥10.8	1280	1.0 (ref)	1.174 (0.886-1.556)	1.014 (0.776-1.396)	1.369 (0.932-2.012)	
Lactate (mmol/L)						0.499
<1.3	409	1.0 (ref)	0.913 (0.510-1.635)	0.725 (0.403-1.303)	0.766 (0.394-1.486)	
≥1.3	2075	1.0 (ref)	1.042 (0.818-1.327)	0.942 (0.742-1.195)	1.238 (0.968-1.583)	
INR						0.110
<1.2	898	1.0 (ref)	0.782 (0.529-1.157)	0.796 (0.533-1.189)	1.108 (0.667-1.841)	
≥1.2	1586	1.0 (ref)	1.052 (0.802-1.379)	0.874 (0.661-1.157)	1.120 (0.768-1.634)	
RDW						0.552
<15.4	1206	1.0 (ref)	0.896 (0.657-1.220)	0.735 (0.529-1.022)	0.741 (0.472-1.162)	
≥15.4	1278	1.0 (ref)	1.014 (0.729-1.409)	1.028 (0.735-1.438)	1.678 (1.089-2.585)	
WBC (10^9^/L)						0.091
<11.9	1200	1.0 (ref)	0.825 (0.594-1.145)	0.802 (0.570-1.128)	1.116 (0.720-1.730)	
≥11.9	1284	1.0 (ref)	1.149 (0.850-1.553)	0.917 (0.673-1.248)	1.245 (0.818-1.894)	
Chronic pulmonary						0.906
Yes	1889	1.0 (ref)	0.954 (0.585-1.554)	0.963 (0.580-1.598)	1.283 (0.674-2.442)	
No	595	1.0 (ref)	0.922 (0.717-1.185)	0.840 (0.647-1.090)	1.084 (0.767-1.531)	
Cardiac arrhythmias						0.095
Yes	1066	1.0 (ref)	0.735 (0.520-1.038)	0.754 (0.529-1.076)	1.251 (0.789-1.983)	
No	1418	1.0 (ref)	1.176 (0.879-1.573)	0.988 (0.731-1.335)	1.162 (0.776-1.739)	
Coagulopathy						0.628
Yes	1710	1.0 (ref)	0.783 (0.521-1.177)	0.805 (0.534-1.213)	1.109 (0.639-1.922)	
No	774	1.0 (ref)	1.098 (0.839-1.438)	0.971 (0.733-1.286)	1.300 (0.892-1.894)	
Congestive heart failure						0.352
Yes	951	1.0 (ref)	0.791 (0.541-1.156)	0.876 (0.596-1.287)	1.261 (0.739-2.154)	
No	1533	1.0 (ref)	0.968 (0.735-1.276)	0.856 (0.643-1.141)	1.069 (0.744-1.538)	
Diabetes						0.748
Yes	601	1.0 (ref)	0.845 (0.487-1.474)	1.243 (0.726-2.127)	1.368 (0.686-2.727)	
No	1883	1.0 (ref)	0.987 (0.773-1.260)	0.862 (0.667-1.113)	1.100 (0.781-1.549)	
Fluid electrolyte						0.621
Yes	1034	1.0 (ref)	0.846 (0.628-1.147)	0.785 (0.578-1.066)	1.006 (0.672-1.507)	
No	1450	1.0 (ref)	1.142 (0.820-1.591)	1.060 (0.750-1.496)	1.462 (0.925-2.312)	
Hypertension						0.812
Yes	1313	1.0 (ref)	1.085 (0.795-1.481)	1.017 (0.737-1.404)	1.528 (0.995-2.348)	
No	1171	1.0 (ref)	0.922 (0.666-1.275)	0.904 (0.644-1.268)	1.038 (0.660-1.632)	
Liver disease						0.792
Yes	542	1.0 (ref)	0.924 (0.582-1.468)	0.999 (0.619-1.611)	1.093 (0.596-2.006)	
No	1942	1.0 (ref)	0.992 (0.766-1.284)	0.891 (0.682-1.164)	1.239 (0.862-1.783)	
Renal failure						0.299
Yes	616	1.0 (ref)	0.906 (0.624-1.315)	0.785 (0.521-1.182)	1.049 (0.599-1.835)	
No	1868	1.0 (ref)	0.963 (0.727-1.275)	1.036 (0.781-1.374)	1.402 (0.967-2.034)	

BCR: blood urea nitrogen/creatinine ratio; RRT: renal replacement therapy; CHF: congestive heart failure; RDW: red blood cell distribution width; INR: international normal ratio; WBC: white blood cell count; SOFA: Sequential Organ Failure Assessment score.

## Data Availability

The datasets used and/or analyzed during the present study were availed by the corresponding author on reasonable request.

## Abbreviations

BCR: Blood urea nitrogen/creatinine ratio
ROC: Receiver operator characteristic curves
SOFA: Sequential Organ Failure Assessment score
APSIII: Acute Physiology Score III
BUN: Blood urea nitrogen
Cr: Creatinine
CHF: Congestive heart failure
PT: Prothrombin time
PTT: Partial thrombin time
RDW: Red blood cell distribution width
INR: International normal ratio
WBC: White blood cell count
MBP: Mean blood pressure
RRT: Renal replacement therapy
BMI: Body mass index
HRs: Hazard ratios
CIs: Confidence intervals
ICU: Intensive care unit
LOS: Length of stay
AUCs: Areas under the ROC curves.
